# Association between parental consumer attitudes with their children’s sensory taste preferences as well as their food choice

**DOI:** 10.1371/journal.pone.0200413

**Published:** 2018-08-01

**Authors:** Hannah S. Jilani, Hermann Pohlabeln, Kirsten Buchecker, Wencke Gwozdz, Stefaan De Henauw, Gabriele Eiben, Dénes Molnar, Luis A. Moreno, Valeria Pala, Lucia Reisch, Paola Russo, Toomas Veidebaum, Wolfgang Ahrens, Antje Hebestreit, on behalf of the IDEFICS consortium

**Affiliations:** 1 Leibniz Institute for Prevention Research and Epidemiology - BIPS, Bremen, Germany; 2 Institute for Public Health and Nursing Research, University of Bremen and Health Sciences Bremen, University of Bremen, Bremen, Germany; 3 Department of Food Science, TTZ, Bremerhaven, Germany; 4 Department of Management, Society and Communication, Copenhagen Business School, Frederiksberg, Denmark; 5 Department of Public Health, Faculty of Medicine and Health Sciences, Ghent University, Ghent, Belgium; 6 Department of Public Health and Community Medicine, University of Gothenburg, Gothenburg, Sweden; 7 Department of Biomedicine and Public Health, School of Health and Education, University of Skövde, Skövde, Sweden; 8 Department of Pediatrics, University of Pécs, Medical School, Pécs, Hungary; 9 GENUD (Growth, Exercise, Nutrition and Development) Research Group, Faculty of Health Sciences, University of Zaragoza, Zaragoza, Spain; 10 Department of Preventive and Predictive Medicine, Fondazione IRCCS Istituto Nazionale dei Tumori, Milan, Italy; 11 Epidemiology and Population Genetics, Institute of Food Sciences, National Research Council, Avellino, Italy; 12 Department of Chronic Diseases, National Institute for Health Development, Tallinn, Estonia; 13 Institute of Statistics, Faculty of Mathematics and Computer Science, University of Bremen, Bremen, Germany; University Sains Malaysia, MALAYSIA

## Abstract

**Background:**

We investigated the association between the consumer attitudes of European parents and their children’s taste preferences and food choice. Furthermore, we studied whether the parental consumer attitudes were related to education level.

**Methods:**

This analysis included 1,407 IDEFICS study children aged 6.0 to 11.8 years and from 7 European countries, who participated in the sensory taste perception module between 2007 and 2010. Parental consumer attitude was operationalized as *‘trusting in foods known from advertisements’ (trusting advertisements)* and as ‘*not avoiding additives in food’ (not avoiding additives)*. Parents reported their educational attainment and completed a food frequency questionnaire for their children. Consumption frequencies of sweet, fatty and processed foods as well as a healthy diet adherence score were calculated. Children performed fat, sweet and umami taste preference tests. Multivariable logistic models were used to analyse the association between *parental consumer attitudes* and their children’s taste preference frequencies as well as parental education. Linear regression models were used to analyse the association between *parental consumer attitudes* and their children’s food consumption.

**Results:**

Parental consumer attitudes were not associated with children’s fat, sweet and umami taste preferences. Children of parents *trusting advertisements* consumed more frequently processed foods (β = 1.21, 95% CI: 0.49; 1.93). Children of parents *not avoiding additives* consumed more often sweet, fatty and processed foods and had a lower healthy diet adherence score (β = 2.37, 95% CI: 1.03; 3.70; β = 2.27, 95% CI: 1.12; 3.43; β = 0.91, 95% CI: 0.22; 1.59; β = -2.87, 95% CI: -3.89; -1.85, respectively). *Unfavourable parental consumer attitudes* were associated with a lower parental education level across Europe (Compared to high education: Odds Ratio (OR) of *trusting advertisements* with medium education: 1.04, 95% CI: 0.77; 1.40; OR with low education: 2.01, 95% CI: 1.15; 3.54; OR of *not avoiding additives* with medium education: 1.91, 95% CI: 1.44; 2.54; OR with low education: 1.76, 95% CI: 0.96; 3.24).

**Conclusions:**

Across Europe, unfavourable parental consumer attitudes are associated with a lower diet quality of their children. Parental consumer attitudes in turn were associated with their own level of education. This has implications for policy makers, interventions and health promotion programmes that aim to promote healthy eating.

## Introduction

A diet high in sugar and fat has been found to contribute to a positive energy balance and unfavourable weight development in children [1]. As parents provide most of the foods children consume, they have a great influence on their children’s diet through their own nutritional behaviour and child-feeding practices [2, 3]. Thus the consumer behaviour of the parents, such as buying particular foods and beverages, may determine their children’s diet as they act as gatekeepers [3]. Parental consumer behaviour can be described as (i) parental attitudes towards food and beverage products they know from advertisements and (ii) parental efforts to avoid additives in food they purchase for the family. These two factors might influence the taste preferences and food consumption frequencies of their children through their gatekeeping function as already mentioned. Foods and beverages that are heavily marketed are typically high in sugar and fat [4] and are thus more unhealthy and more obesogenic. Furthermore, literature describes that foods containing additives, including taste enhancers, preservatives, and colours might be harmful for children [5].

It is unclear if these described attitudes, hereafter called parental consumer attitudes, are associated with children’s taste preferences as well as their sweet, fatty and processed food consumption frequency, and the overall quality of their diet. During childhood food choice is mostly determined by the sensory perception and liking of foods [6, 7]. Sensory taste perception and preferences formed during childhood might persist until later life [8] and affect dietary intake. Hence, to influence food choice of young children it is essential to understand how taste perception and preferences are formed. It is also unclear to what extent the parental consumer attitudes are related to their educational level. If there is no other access to nutritional knowledge, parents with a lower education might be more receptive for what is said in advertisements and might hence put more trust in the information given therein.

To understand what influences the dietary behaviour of children, it is essential to investigate how taste and food preferences are formed. Therefore, we investigated whether parental consumer attitudes are associated with parental educational level as well as with their children’s taste preferences and food consumption patterns. Should these associations prove to be present, this could be a pathway from low educational level to an unhealthy diet and thus offer a starting point for interventions.

## Methods

### Subjects

The data for this study was taken from the IDEFICS (Identification and prevention of Dietary- and lifestyle-induced health EFfects In Children and infantS) multicentre study, which aimed to investigate the prevalence and aetiology of overweight and obesity of children in Europe and to implement and evaluate intervention programs to reduce childhood overweight and unhealthy lifestyles [9]. Between September 2007 and June 2008, a baseline survey (T0) was conducted on 16,228 aged two to 9.9 year old children from 8 European countries (Belgium, Cyprus, Estonia, Germany, Hungary, Italy, Spain and Sweden). Two years later, between September 2009 and May 2010 a follow up (T1) examination was conducted on 13,596 children. The children were approached through primary school and kindergarten settings. A sub-sample of 20% of schoolchildren from the age of 6 years onwards was asked to participate in taste preference tests. In total, 1,407 children between 6.0 and 11.8 years from Belgium, Estonia, Germany, Hungary, Italy, Spain and Sweden who participated in the preference tests either at T0 or T1 were included in this cross-sectional analysis.

All centres obtained ethical approval from their respective local institutional review board (e.g. Ethics Committee, University Hospital, Gent, Belgium; Tallinn Medical Research Ethics Committee, Tallinn, Estonia; Ethics Committee of the University of Bremen, Bremen, Germany; Egészségügyi Tudományos Tanács, Pécs, Hungary; Azienda nitaria Locale Avellino Comitato Etico, Avellino, Italy; Regionala Etikprövningsnämnden i Göteborg, Gothenburg, Sweden; Comité Ético de Investigación Clínica de Aragón, Zaragoza, Spain). All parents gave informed written consent for their children’s participation. The children were informed orally about the study procedures and gave their oral consent before participating. All instruments, questionnaires and recording sheets were developed in English, translated to the national language and then back-translated to check for translation errors. National examples of foods or other items were provided where deemed necessary.

### Parental consumer attitudes

The children’s dietary behaviour and the parental consumer attitudes regarding nutrition were assessed using the Children’s Eating Habit Questionnaire (CEHQ) [10, 11]. Among others, the parents were asked whether they *trust in products that they know from advertisements* and whether they *try to avoid additives in foods and beverages*. They could answer 5 ‘agree’, 4 ‘agree moderately’, 3 ‘unsure’, 2 ‘disagree moderately’ or 1 ‘disagree’. Parents that answered with ‘unsure’ were excluded from the present analysis. The answering categories of these two variables were dichotomised as: ‘Do not trust in products from advertisements’ (answers: disagree and moderately disagree) vs. ‘Trust in products from advertisements’ (answers: agree and agree moderately) and ‘Do not avoid additives in foods and beverages’ (answers: disagree and moderately disagree) vs. ‘Avoid additives in foods and beverages’ (answers: agree and agree moderately). To ease reading of the text, the following shorter terms will be used henceforth in the text: ‘trust in products from advertisements’ will be stated as ‘trusting advertisements’ and ‘do not avoid additives in foods and beverages’ will be stated as ‘not avoiding additives’

### Parental education

Self-reported educational attainment was classified according to the International Standard Classification of Education (ISCED). The ISCED classification comprises levels 0–8 (ISCED level 0: Early childhood education, ISCED level 1: Primary education, ISCED level 2: Lower secondary education, ISCED level 3: Upper secondary education, ISCED level 4: Post-secondary non-tertiary education, ISCED level 5: Short-cycle tertiary education, ISCED level 6: Bachelor’s or equivalent level, ISCED level 7: Master’s or equivalent level, ISCED level 8: Doctoral or equivalent level) [12]. For the present analysis, highest education level of the household was clustered into three groups; ‘Low’ (ISCED level 0–2), ‘medium’ (ISCED level 3–5) and ‘high’ (ISCED level 6–8).

### Sensory taste preferences

As children were very young and the examination protocol was very demanding, we decided to keep the test procedures as simple as possible. Thus, the whole test procedure was standardised and adapted for use in very young children. We conducted extensive pre-tests and found that the test procedures were only suitable for children from the age of 6 years and not for pre-schoolers [13]. Therefore, the minimum age was set to 6 years during the actual taste threshold tests. Children from the age of 6 years and older were in general able to understand the task and to deliver reliable results. Nevertheless, the procedure needed to be simple and comprehensive. A paired comparison forced choice taste preference test to assess the sweet, salty, umami, aroma and fatty taste preference was arranged as a board game as described in Knof et al. [14]. In brief, for every taste modality, two food samples were presented to the child at room temperature. Apple juice was used to assess sweet and aroma preference and crackers were used to assess salty, fatty and umami preference. One sample represented the basic taste and the other the modified taste (e.g. 3.11% sucrose, 0.05% apple aroma, 1.16% sodium chloride, 18% fat or 1% monosodium glutamate were added). Samples were placed at the bottom of the game board and the child compared the basic sample against the modified one. The interviewer asked the child which of the two samples he/she liked best. The child indicated this by placing the preferred sample on the respective field on the game board. The child had to choose the preferred sample and was then classified as preferring either the basic or the modified taste. The interviewer recorded the answer on paper recording sheets. After each taste modality, participants had a 2-minute break during which they neutralised their palate with distilled water, and the interviewer prepared the next test sequence. According to the examination protocol, at least 1 hour had passed after having eaten but children were not hungry either. The adapted procedures were adjusted after being pre-tested in all survey centres [13]. A test-retest analysis of the taste preference tests in a sub-sample of participating children revealed a kappa coefficient of 0.77 (sweet), 0.86 (salty), 0.77 (fatty), 0.78 (aroma) and 0.80 (umami). The results of the test re-test procedure in the sub-sample were assumed to be applicable to the full sample. The samples were shipped centrally for examination and standard operation protocols (SOP) as well as a central training of field staff were provided to all centres. To ensure a high degree of standardisation, the adherence to the SOPs was monitored during site visits.

In this analysis we investigated the sweet, fat and umami taste preferences as dichotomous variables (basic taste preference vs. modified taste preference).

### Food choices and healthy diet score

As part of the CEHQ, parents were asked to complete a proxy food frequency questionnaire (FFQ) for their children’s diet during the previous month. It was tested for reliability and validity [11, 15]. The questionnaire had 43 items including processed, fatty and sweet, as well as healthy foods and beverages. The answer possibilities were ‘never/less than once a week', ‘1–3 times a week’, ‘4–6 times a week’, ‘1 time/day’, ‘2 times a day’, ‘3 times a day’ and ‘I have no idea’. Based on these answers, the children’s weekly consumption frequencies were calculated. The relative consumption frequency of fatty (fried potatoes, whole fat milk, whole fat yoghurt, fried fish, cold cuts/sausages, fried meat, fried eggs, mayonnaise, cheese, chocolate- or nut-based spread, butter/margarine on bread, nuts/seed/dried fruits, salty snacks, savoury pastries, chocolate-based candies, cake/pudding/cookies and ice cream) as well as sweet (fresh fruits with added sugar, fruit juice, sugar-sweetened drinks, sweetened breakfast cereals, sweetened milk, sweetened yoghurt, jam/honey, chocolate- or nut-based spread, chocolate-based candies, non-fat candies, cake/pudding/cookies and ice cream) foods and beverages per week over all foods were calculated, resulting in the continuous variables sweet propensity score and fat propensity score ranging from 0% to 100% [16]. The consumption frequency of processed foods was calculated as the sum of weekly intake of processed meat, hamburgers, hot dogs, kebab and snacks such as savoury pastries and fritters reported for the previous month.

Based on weekly consumption frequencies, an a priori healthy diet adherence score (HDAS) was calculated [17]. The HDAS aimed to reflect the adherence of the children to food-based dietary guidelines common in all participating countries. In particular: limited intake of refined sugar, reduced fat intake, choosing whole meal foods, high consumption of fruits and vegetables and consuming 2–3 times fish per week. Hence, the HDAS consisted of 5 dimensions including weekly consumption frequency of foods high in sugar, of foods low in fat, of whole meal foods, of fruits and vegetables as well as of fish. Depending on the consumption frequency derived from the FFQ for each dimension, a score between 0 and 10 could be reached. To calculate this, the consumption frequency of foods high in sugar, of foods low in fat, of whole meal foods, of fruits and vegetables as well as of fish, were considered either as total consumption frequency or as the proportion of all consumed food items as follows: To obtain a score for each dimension

1. The weekly consumption frequency of sugar containing foods was divided by the weekly consumption frequency of all consumed foods. The following food items were considered as sugar containing foods: sweetened breakfast cereals, sweetened drinks, fruit juices, sweetened milk, sweet yoghurt and fermented milk beverages, fruits with added sugar, jam and honey, chocolate or nut based chocolate, candy bars, loose candies, marshmallows, biscuits, cakes, pastries, puddings, ice cream, milk or fruit based bars. A proportion of 10% or less of sugar containing foods was assigned to the score of 10, and a proportion of 15.7% or more was assigned to 0. The scores of 1 to 9 were equally distributed in steps of 0.6 percentage points.
2. The weekly consumption frequency of foods generally low in fat (cooked vegetables, eggs, fish, meat and low fat dairy products and spread) was divided by the intake of both cooked and fried vegetables, eggs, fish, meat and low and high fat dairy products and spread. The proportion of foods low in fat over all fat containing food items was assigned to scores of 1 to 10 by assigning 100% low fat foods to 10 and 10% or less to 0. The scores 1 to 9 were equally distributed in steps of 10%.
3. The proportion of whole meal bread (‘whole meal bread, dark roll, dark crispbread’) over all kinds of bread (‘white bread, white roll, white crispbread’ + ‘whole meal bread, dark roll, dark crispbread’) was calculated. A proportion of 100% whole meal bread was assigned to 10 and a proportion of 10% or less to 0. The scores 1 to 9 were equally distributed in steps of 10%.
4. The total consumption frequency of fruits and vegetables (fresh fruits, cooked vegetables, potatoes and beans, legumes and raw vegetables) of 35 portions per week (5 a day) was assigned to a score of 10. Less than 1 portion per week was assigned to 0. The scores 1 to 9 were equally distributed in equidistant categories.
5. The total consumption frequency of fish of 2.5 times per week was assigned to a score of 10. No fish at all was assigned to 0. The scores 1 to 9 were equally distributed in steps of 0.3 times per week.

For the total HDAS, the scores of all dimensions were summed up. Thus, the HDAS ranged from 0 to 50. A higher score represented a higher adherence to the food-based dietary guidelines, presumed to be a healthier diet.

### Potential confounders

Children’s age, sex and country were assessed via parental questionnaires. Children’s weight and height were measured in an overnight fasting state using a Tanita BC 420 SMA scale (TANITA, Tokyo, Japan) for weight measurement and a SECA 225 Stadiometer (SECA GmbH & KG, Hamburg, Germany) for height measurement. BMI was calculated and converted to age- and sex-specific z-scores. Children were classified into thin/normal weight and overweight/obese (weight status) using age- and sex-specific cut-points published by Cole and Lobstein [18]. Children’s age, sex, country and weight status were considered as possible confounders.

### Statistical analysis

The associations between the two dichotomised variables for parental consumer attitudes and the dichotomous outcome variables children’s sweet, fat and umami taste preferences were estimated using logistic regression models. Models were adjusted for sex, age, country, weight status and highest parental education level as fixed effects.

The associations between parental consumer attitudes and the continuous outcome variables fatty, sweet as well as processed food consumption and HDAS of their children were analysed using linear regression models adjusted for sex, age, country, weight status and highest parental education level as fixed effects. Sample sizes for these analyses varied due to missing values for the avoiding additives variable (missing: n = 61).

Further, the association between highest parental education level and parental consumer attitudes was analysed by means of a logistic regression (adjusted only for country but not for child dependent variables).

We used SAS^®^ (Statisitcal Analysis System, SAS Institute Inc., Cary, USA), version 9.3 for all analyses and set the level of significance at p<0.05.

## Results

### Study characteristics

The study sample comprised 689 girls (49.0%) and 718 boys (51.0%). The age ranged from 6.0 to 11.8 years (mean 8.8 years). 22.4% of the children were overweight or obese. 5.5% of the children had parents with low education, 42.9% with medium education and 51.6% with high education.

About 56% and 58.6% of all children preferred the test sample with the added fat and added sugar over the basic taste sample, respectively, and 45% of all children preferred the sample with added monosodium glutamate over the sample without added monosodium glutamate. Regarding parents, 21.8% stated that they trust in foods and beverages they know from advertisements and 26.2% answered that they do not avoid foods and beverages with additives. Children consumed processed food products on average 6.4 times per week and had fatty and sweet food and beverage propensity scores of 26.4% and 25.4%, respectively. There were no substantial differences between boys and girls with regard to the study characteristics (Table 1).

**Table 1:** Characteristics (total number and percentages or mean and standard deviation (SD)) of exposure and outcome variables given by boys and girls.

	Boys	Girls	Total
	N = 718 (51.0%)	N = 689 (49.0%)	N = 1407
	**Mean (SD)**	**Mean (SD)**	**Mean (SD)**
**Age**	8.8 (1.2)	8.8 (1.1)	8.8 (1.1)
**Sweet propensity score %**	26.0 (11.4)	24.8 (10.4)	25.4 (11.0)
**Fat propensity score core %**	26.3 (9.4)	26.5 (9.6)	26.4 (9.5)
**Processed foods/week**	6.4 (5.6)	6.4 (6.1)	6.4 (5.8)
**Healthy diet adherence score**	21.7 (8.5)	21.9 (8.4)	21.8 (8.5)
	**N (%)**	**N (%)**	**N (%)**
**Overweight/obese**^1^	147 (20.5)	168 (24.4)	315 (22.4)
**Parents who trust in advertisements**	153 (21.3)	154 (22.4)	307 (21.8)
**Parents who do not avoid additives**	172 (24.9)	180 (27.5)	352 (26.2)
**Highest parental education level**^2^			
Low	44 (6.1)	33 (4.8)	77 (5.5)
Medium	294 (41.0)	310 (45.0)	604 (42.9)
High	380 (52.9)	346 (50.2)	726 (51.6)
**Sweet preference**	427 (59.5)	398 (57.8)	825 (58.6)
**Fat preference**	399 (55.6)	387 (56.2)	786 (55.9)
**Umami preference**	332 (46.2)	301 (43.7)	633 (45.0)
**Country**			
Belgium	106 (14.8)	94 (13.6)	200 (14.2)
Estonia	66 (9.2)	72 (10.5)	138 (9.8)
Germany	205 (28.6)	221 (32.1)	426 (30.3)
Hungary	96 (13.4)	92 (13.4)	188 (13.4)
Italy	84 (11.7)	71 (10.3)	155 (11.0)
Spain	98 (13.7)	76 (13.4)	174 (12.4)
Sweden	63 (8.8)	63 (9.1)	126 (9.0)

^1^: BMI z-scores according to Cole and Lobstein 2012 [18].

^2^: International Standard Classification of Education Maximum (ISCED) [12]; maximum of both parents (0, 1, 2 = low education; 3, 4 = medium education; 5, 6 = high education).

### Parental consumer attitudes and children’s taste preferences

In general, country, sex, age, weight status and parental education were significant confounders (p<0.05) in almost all analyses (data not shown). For example, boys consumed sweet foods more frequently (p<0.05) and increasing age was associated with increasing consumption frequency of processed foods (p<0.05). However, since these influences are beyond the scope of the present analysis, we will focus our attention on the results presented hereafter which are relevant for our study aim.

The logistic regression analysis revealed no association between trusting advertisements and children’s sweet, fat or umami preferences (Table 2). Further, we did not observe an association between not avoiding additives and children’s sweet, fat or umami preferences.

**Table 2:** Association between parental consumer attitudes and their children’s taste preferences^1^.

Outcome:	Parents trust in advertisements^2^							Parents do not avoid additives^3^					
	No		Yes					No		Yes			
	N	%	N	%	OR (95% CI)	p-value		N	%	N	%	OR (95% CI)	p-value
**Sweet preference**													
Low	462	42.0	120	39.1	1.00 (reference)			163	46.3	395	39.7	1.00 (reference)	
High	638	58.0	187	60.9	0.94 (0.71; 1.25)	0.69		189	53.7	599	60.3	0.85 (0.65; 1.11)	0.23
**Fat preference**													
Low	495	45.0	126	41.0	1.00 (reference)			442	44.5	156	44.3	1.00 (reference)	
High	605	55.0	181	59.0	1.07 (0.81; 1.42)	0.64		552	55.5	196	55.7	1.01 (0.78; 1.32)	0.92
**Umami preference**													
Low	609	55.4	165	53.8	1.00 (reference)			545	54.8	193	54.8	1.00 (reference)	
High	491	44.6	142	46.2	1.02 (0.77; 1.35)	0.89		449	45.2	159	45.2	1.04 (0.80; 1.36)	0.78
**All children**	1407							1346					

Abbreviations: OR = Odds Ratio, CI = Confidence interval

^1^: Logistic regression model, adjusted for sex, age, country, ISCED (International Standard Classification of Education Maximum (ISCED) [12]) and weight status [18]

^2^: Reference: Parents do not trust in advertisement

^3^: Reference: Parents avoid additives

### Parental consumer attitudes and children’s food choice

We observed a non-significant but positive association between parental trusting advertisements and children’s sweet (ß = 0.97, 95% CI = -0.45; 2.40) and fatty (ß = 0.79, 95% CI = -0.43; 2.00) food and beverage consumption. Further, we found a significantly higher consumption frequency of processed foods (ß = 1.21, 95% CI = 0.49; 1.93) in children whose parents trust advertisements than in those whose parents do not trust advertisements. Children of parents not avoiding additives consumed sweet and fatty foods and beverages over all foods more often and also consumed processed foods more often per week (ß = 2.37, 95% CI = 1.03; 3.70; ß = 2.27, 95% CI = 1.12; 3.43; ß = 0.91, 95% CI = 0.22; 1.59, respectively) compared to children whose parents avoided additives. Children whose parents did not avoid additives also had a significantly lower HDAS (ß = -2.87, 95% CI = -3.89; -1.85) compared to those whose parents avoided additives (Table 3).

**Table 3:** Association between parental consumer attitudes and their children’s food consumption^1^.

	Parents trust in advertisements^2^		Parents do not avoid additives^3^	
Outcome:	ß (95% CI)	p-value	ß (95% CI)	p-value
**Proportion of sweet food and beverage consumption**	0.97 (-0.45; 2.40)	0.18	2.37 (1.03; 3.70)	0.0005
**Proportion of fatty food consumption**	0.79 (-0.43; 2.00)	0.21	2.27 (1.12; 3.43)	0.0001
**Processed food consumption frequency**	1.21 (0.49; 1.93)	0.001	0.91 (0.22; 1.59)	0.0093
**Healthy Diet Adherence Score**	-0.42 (-1.50; 0.67)	0.45	-2.87 (-3.89; -1.85)	<0.0001

Abbreviations: ß = ß-coefficient, CI = Confidence interval

^1^: Model is adjusted for sex, age, country, ISCED (International Standard Classification of Education Maximum (ISCED) [12]) and weight status.

^2^: Reference: Parents do not trust in advertisements.

^3^: Reference: Parents avoid additives.

### Parental education level and consumer attitudes

Highest parental education level was associated with parental consumer attitudes. Parents with low education level had significant higher odds (OR = 2.01, 95% CI = 1.15; 3.54) of trusting advertisements compared to parents with high education level. Further, parents with medium or low education level had higher odds (OR = 1.91, 95% CI = 1.44; 2.54 and OR = 1.76 95% CI = 0.96; 3.24, respectively) of not avoiding additives compared to parents with high education level (Table 4).

**Table 4:** Influence of parental ISCED level on their consumer attitudes^1^.

	High ISCED level (Reference)		Medium ISCED level				Low ISCED level			
Outcome	N	%	N	%	OR (95% CI)	p-value	N	%	OR (95% CI)	p-value
**Parents trust in advertisement**										
No	600	82.6	456	75.5	1.00 (reference)		44	57.1	1.00 (reference)	
Yes	126	17.4	148	24.5	1.04 (0.77;1.40)	0.81	33	42.9	2.01 (1.15;3.54)	0.02
**Parents do not avoid additives**										
No	539	76.9	403	70.2	1.00 (reference)		52	73.2	1.00 (reference)	
Yes	162	23.1	171	29.8	1.91 (1.44;2.54)	<0.0001	19	26.8	1.76 (0.96;3.24)	0.07

Abbreviation: OR: Odds ratio, CI: Confidence interval, ISCED: International Standard Classification of Education Maximum (ISCED) [12]; maximum of both parents (0, 1, 2 = low education; 3, 4, 5 = medium education; 6, 7, 8 = high education)

^1^: Model is adjusted for country.

## Discussion

Our analyses revealed that parental consumer attitudes are associated with their children’s (parental reported) food intake but not with their taste preferences, and that the parental attitudes depend on parental education level across Europe.

We did not find any association between parental consumer attitudes and general preferences for sweet, fat and umami. Therefore, the diet that is associated with the parental consumer attitudes did not appear to be related to taste preferences. Results of a previous study, also suggested that taste preferences might not be associated with dietary habits [16]. We presume that taste preferences might be determined by other factors and that children do not necessarily prefer what parents provide. Furthermore, using food matrices for the taste preference tests limited the textual conditions of foods to only juice and crackers, compared to the variety of textures children were exposed normally. This may have caused the non-significant association between parental consumer attitudes and taste preferences. The perception of taste may differ according to the matrix delivering the taste in the oral cavity. For example, fat in more liquid foods might be more ready to diffuse out compared to solid food matrices like crackers. Accordingly, a semi-fluid matrix possibly allows the sweet taste to remain longer in the oral cavity compared to apple juice. This should be considered in future studies. We observed that children from parents trusting advertisements tended to consume fatty and sweet foods and beverages more often and also consumed more processed foods. These children had a non-significant tendency to have a lower diet quality indicated by a lower HDAS. Previous studies in the USA and in Europe showed that 20% of commercials shown on TV promote food products and 98% of these advertisements promote foods and beverages high in sugar, sodium and/or fat [19–21]. As a result, the parents trusting advertisements may prefer to buy the advertised products, which may explain the tendency towards a lower overall quality of the diet and a higher consumption frequency of sweet and fatty as well as processed foods and beverages.

Even more pronounced was the effect of parental consumer attitude on their children’s dietary behaviour when considering the analyses of the associations of not avoiding additives. Children of parents not avoiding additives had a lower overall diet quality, consumed sweet foods and beverages more often, and also consumed more fatty foods as well as more processed foods. A recent study from Norway found a negative association between a consumer pattern labelled as ‘healthy’ which included the importance of absence of additives and junk/convenience food consumption [22]. They concluded that healthy eating promotion of children should focus on parents’ health concerns, emphasising on natural food without additives.

We found that a lower parental education (from high over medium to low) is associated with unfavourable consumer attitudes (trusting advertisements and not avoiding). Since children with less educated parents have a lower overall diet quality and consume more sweet and fatty foods [23], our results add valuable knowledge about the pathway from low education to an unfavourable diet, indicating that families with a lower socioeconomic status consume unhealthy and more obesogenic food, because they tend to rely on advertisements more often and do not try to avoid additives.

Because early learned dietary behaviour and taste preferences can persist until later on in life [8], it very important to ensure a healthy and adequate diet during childhood, that is particularly low in sugar and fat, so as to prevent an early onset of overweight. To be able to do so, parents need information about a healthy diet for their children.

This study has some strengths and limitations that need to be addressed. As parents reported the dietary intake of their children, underreporting could have occurred due to social desirability and to missing details on their children’s outside home eating. Nevertheless, self-reported food group consumption is more robust against misreporting compared to energy reporting [24]. The described underreporting would have led to a decrease of the association between parental consumer attitudes and children’s food intake. The same holds true for the misreporting of consumer attitudes due to social desirability. Should parents have reported more favourable consumer attitudes, this could have also led to an attenuation of the association we investigated. Apart from these limitations our study has several strengths. All measurements including the taste preference tests were developed especially for children. A high standardisation between countries was assured through the central training sessions offered to all study personnel and the central provision of all study materials. Site visits further guaranteed high quality of data collection. All methods were pre-tested and test-retest reliability was calculated with ‘almost perfect’ results [13, 14]. Our study sample consists of a large number of children and we were able to account for relevant possible confounders.

To our knowledge, this is the first study that investigated the impact of parental consumer attitudes on their children’s taste preferences and dietary habits in different European countries using standardised methods. Our results can possibly to some extent explain the higher prevalence of overweight and obesity [25–27] as well as the higher consumption of sweet and fatty foods [23, 28] in children from families with a lower socioeconomic status.

## Conclusion

Our results show that parents’ consumer attitudes are associated with their children’s food intake. Additionally, we showed that parents with a low education background have less favourable consumer attitudes. This has implications for policy makers, interventions and health promotion programmes that aim to promote healthy eating. Additionally, our results underline the importance of an incorporation of nutrition and consumer education into school education to help develop a healthier dietary as well as consumer behaviour among children.

## Supporting information

S1 File Full author list of IDEFICS consortium. (DOCX)

## References

[1] Pala V, Lissner L, Hebestreit A, Lanfer A, Sieri S, Siani A, et al Dietary patterns and longitudinal change in body mass in European children: a follow-up study on the IDEFICS multicenter cohort. Eur J Clin Nutr. 2013;67(10):1042–9. Epub 2013/08/15. 10.1038/ejcn.2013.145 . 23942180

[2] Birch LL. Psychological influences on the childhood diet. J Nutr. 1998;128(2 Suppl):407S–10S. Epub 1998/03/21. 10.1093/jn/128.2.407S . 9478037

[3] John DR. Consumer socialization of children: A retrospective look at twenty-five years of research. *Journal of Consumer Research*. 1999;26:183–213.

[4] Batada A, Seitz MD, Wootan MG, Story M. Nine out of 10 food advertisements shown during Saturday morning children’s television programming are for foods high in fat, sodium, or added sugars, or low in nutrients. J Am Diet Assoc. 2008;108(4):673–8. Epub 2008/04/01. 10.1016/j.jada.2008.01.015 . 18375225

[5] McCann D, Barrett A, Cooper A, Crumpler D, Dalen L, Grimshaw K, et al Food additives and hyperactive behaviour in 3-year-old and 8/9-year-old children in the community: a randomised, double-blinded, placebo-controlled trial. Lancet. 2007;370(9598):1560–7. Epub 2007/09/11. 10.1016/S0140-6736(07)61306-3 . 17825405

[6] Birch LL, Fisher JO. Development of eating behaviors among children and adolescents. Pediatrics. 1998;101(3 Pt 2):539–49. . 12224660

[7] Glanz K, Basil M, Maibach E, Goldberg J, Snyder D. Why Americans eat what they do: taste, nutrition, cost, convenience, and weight control concerns as influences on food consumption. J Am Diet Assoc. 1998;98(10):1118–26. 10.1016/S0002-8223(98)00260-0 . 9787717

[8] Skinner JD, Carruth BR, Bounds W, Ziegler P, Reidy K. Do food-related experiences in the first 2 years of life predict dietary variety in school-aged children? J Nutr Educ Behav. 2002;34(6):310–5. Epub 2003/01/31. . 12556269 10.1016/s1499-4046(06)60113-9

[9] Ahrens W, Bammann K, Siani A, Buchecker K, De Henauw S, Iacoviello L, et al The IDEFICS cohort: design, characteristics and participation in the baseline survey. Int J Obes (Lond). 2011;35 Suppl 1:S3–15. Epub 2011/04/22. 10.1038/ijo.2011.30 . 21483420

[10] Scholderer J, Brunso K, Bredahl L, Grunert KG. Cross-cultural validity of the food-related lifestyles instrument (FRL) within Western Europe. Appetite. 2004;42(2):197–211. Epub 2004/03/11. 10.1016/j.appet.2003.11.005 . 15010184

[11] Lanfer A, Hebestreit A, Ahrens W, Krogh V, Sieri S, Lissner L, et al Reproducibility of food consumption frequencies derived from the Children’s Eating Habits Questionnaire used in the IDEFICS study. Int J Obes (Lond). 2011;35 Suppl 1:S61–8. Epub 2011/04/22. 10.1038/ijo.2011.36 . 21483424

[12] UNESCO. International Standard Classification of Education 2012 [cited 2016 27.10.2016]. http://www.uis.unesco.org/Education/Documents/isced-2011-en.pdf.

[13] Suling M, Hebestreit A, Peplies J, Bammann K, Nappo A, Eiben G, et al Design and results of the pretest of the IDEFICS study. Int J Obes (Lond). 2011;35 Suppl 1:S30–44. Epub 2011/04/22. 10.1038/ijo.2011.33 . 21483421

[14] Knof K, Lanfer A, Bildstein MO, Buchecker K, Hilz H. Development of a method to measure sensory perception in children at the European level. Int J Obes (Lond). 2011;35 Suppl 1:S131–6. Epub 2011/04/22. 10.1038/ijo.2011.45 . 21483413

[15] Bel-Serrat S, Mouratidou T, Pala V, Huybrechts I, Bornhorst C, Fernandez-Alvira JM, et al Relative validity of the Children’s Eating Habits Questionnaire-food frequency section among young European children: the IDEFICS Study. Public Health Nutr. 2014;17(2):266–76. Epub 2013/01/05. 10.1017/S1368980012005368 . 23286734 PMC10282422

[16] Lanfer A, Knof K, Barba G, Veidebaum T, Papoutsou S, de Henauw S, et al Taste preferences in association with dietary habits and weight status in European children: results from the IDEFICS study. Int J Obes (Lond). 2012;36(1):27–34. Epub 2011/08/17. 10.1038/ijo.2011.164 . 21844876

[17] Arvidsson L, Eiben G, Hunsberger M, De Bourdeaudhuij I, Molnar D, Jilani H, et al Bidirectional associations between psychosocial well-being and adherence to healthy dietary guidelines in European children: prospective findings from the IDEFICS study. BMC Public Health. 2017;17(1):926 Epub 2017/12/15. 10.1186/s12889-017-4920-5 . 29237434 PMC5729410

[18] Cole TJ, Lobstein T. Extended international (IOTF) body mass index cut-offs for thinness, overweight and obesity. Pediatr Obes. 2012;7(4):284–94. Epub 2012/06/21. 10.1111/j.2047-6310.2012.00064.x . 22715120

[19] Hawkes C. Regulating and litigating in the public interest: regulating food marketing to young people worldwide: trends and policy drivers. Am J Public Health. 2007;97(11):1962–73. Epub 2007/09/29. 10.2105/AJPH.2006.101162 . 17901436 PMC2040356

[20] Gantz W S N.; Angelini J.R.; Rideout V. Food for thought: television food advertising to children in the United States. Kaiser Family Foundation, 2007.

[21] Cairns G A K; Hastings G. The extent, nature and effects of food promotion to children: a review of the evidence to December 2008. World Health Organisation, 2009.

[22] Oellingrath IM, Hersleth M, Svendsen MV. Association between parental motives for food choice and eating patterns of 12- to 13-year-old Norwegian children. Public Health Nutr. 2013;16(11):2023–31. Epub 2012/10/05. 10.1017/S1368980012004430 . 23034288 PMC10271725

[23] Fernandez-Alvira JM, Mouratidou T, Bammann K, Hebestreit A, Barba G, Sieri S, et al Parental education and frequency of food consumption in European children: the IDEFICS study. Public Health Nutr. 2013;16(3):487–98. Epub 2012/06/13. 10.1017/S136898001200290X . 22687743 PMC10271421

[24] Subar AF, Freedman LS, Tooze JA, Kirkpatrick SI, Boushey C, Neuhouser ML, et al Addressing Current Criticism Regarding the Value of Self-Report Dietary Data. J Nutr. 2015;145(12):2639–45. 10.3945/jn.115.219634 . 26468491 PMC4656907

[25] Danielzik S, Czerwinski-Mast M, Langnase K, Dilba B, Muller MJ. Parental overweight, socioeconomic status and high birth weight are the major determinants of overweight and obesity in 5–7 y-old children: baseline data of the Kiel Obesity Prevention Study (KOPS). Int J Obes Relat Metab Disord. 2004;28(11):1494–502. Epub 2004/08/25. 10.1038/sj.ijo.0802756 . 15326465

[26] Bammann K, Gwozdz W, Lanfer A, Barba G, De Henauw S, Eiben G, et al Socioeconomic factors and childhood overweight in Europe: results from the multi-centre IDEFICS study. Pediatr Obes. 2013;8(1):1–12. Epub 2012/08/14. 10.1111/j.2047-6310.2012.00075.x . 22888012

[27] Fernandez-Alvira JM, te Velde SJ, De Bourdeaudhuij I, Bere E, Manios Y, Kovacs E, et al Parental education associations with children’s body composition: mediation effects of energy balance-related behaviors within the ENERGY-project. Int J Behav Nutr Phys Act. 2013;10:80 Epub 2013/06/27. 10.1186/1479-5868-10-80 . 23800170 PMC3695820

[28] Fernandez-Alvira JM, De Bourdeaudhuij I, Singh AS, Vik FN, Manios Y, Kovacs E, et al Clustering of energy balance-related behaviors and parental education in European children: the ENERGY-project. Int J Behav Nutr Phys Act. 2013;10:5 Epub 2013/01/17. 10.1186/1479-5868-10-5 . 23320538 PMC3618064

