# Supplementary material for: Association between parental consumer attitudes with their children’s sensory taste preferences as well as their food choice
Source: PLoS One. 2018 Aug 1;13(8):e0200413. doi: 10.1371/journal.pone.0200413 (PMC6070197; doi:10.1371/journal.pone.0200413)
Supplement: S1 File — (DOCX) [file pone.0200413.s001.docx]

**IDEFICS Consortium Group:**

**General Coordinator**: Wolfgang Ahrens. **Steering Committe**: Wolfgang Ahrens, Iris Pigeot, Karin Bammann, Alfonso Siani, Lauren Lissner, Stefaan De Henauw, Staffan Mårild, Licia Iacoviello, Andreu Palou, Lucia Reisch, Dénes Molnar, Garrath Williams. **Principal investigators**: Wolfgang Ahrens, Stefaan De Henauw, Michael Tornaritis, Dénes Molnár, Luis A. Moreno, Staffan Mårild, Toomas Veidebaum, Lucia Reisch, Michel de Lorgeril, Kirsten Buchecker, Yannis Pitsiladis, Garrath Williams, Licia Iacoviello, Vittorio Krogh, Claudio Galli, Andreu Palou, Laura Fernandez, Arno Fraterman, Mark Rayson, Fernando Giacco, Ralf Gockel. **Partners: Agorà Med srl (Italy):** Fernando Giacco, Riccardo Siani; **BioTel Ltd (UK):** Mark P. Rayson; **Copenhagen Business School (Denmark):** Lucia Reisch, Wencke Gwozdz; **Laboratoriumsmedizin Dortmund, Dr. Eberhard und Partner (Germany):** Arno Fraterman; **European Food Information Council (Belgium):** Laura Fernandez, Stefan Storcksdieck, Josephine Wills; **Fondazione IRCCS Istituto Nazionale Tumori (Italy)**: Vittorio Krogh, Valeria Pala, Sabina Sieri; **Gockel Design (Germany):** Ralf Gockel; **National Research Council, Institute of Food Sciences** (Italy): Alfonso Siani, Gianvincenzo Barba (†), Fabio Lauria, Annunziata Nappo, Paola Russo; **National Institute for Health Development (Estonia)**: Toomas Veidebaum, Kenn Konstabel, Helle-Mai Loit, Marge Saamel; **Research and Education Institute of Child Health (Cyprus)**: Michael Tornaritis, Charalambos Hadjigeorgiou, Christoforos Hadjioannou, Yiannis Kourides, Stalo Papoutsou, Antonia Solea; **Technologie-Transfer-Zentrum (Germany)**: Kirsten Buchecker; **CuoreIRCCS Istituto Neurologico Mediterraneo, Neuromed (Italy ):** Licia Iacoviello, Daniela Cugino, Francesco Gianfagna, Fabrizia Noro, Claudio GrippiIolanda Santimone; **Ghent University (Belgium)**: Stefaan De Henauw, Ilse De Bourdeaudhuij, Ariana Ghekiere, Lea Maes, Nathalie Michels, Charlene Ottevaere, Isabelle Sioen, Barbara Vanaelst, Vera Verbestel; **University of Brighton (Scotland):** Yannis Pitsiladis, Guan Wang; **University of Gothenburg (Sweden)**: Staffan Mårild, Aravinda Berggreen-Clausen, Patrick Bergman, Gabriele Eiben, Monica Hunsberger, Lauren Lissner, Kirsten Mehlig, Stina Olafsdottir, Susan Regber, Gianluca Tognon, Bojan Tubic, Asa Svensson; **University Illes Balears (Spain)**: Andreu Palou, Catalina Picó, Teresa Priego, Joana Sánchez; **Lancaster University (United Kingdom)**: Garrath Williams; **University of Bremen (Germany):** Wolfgang Ahrens, Karin Bammann, Iris Pigeot-Kuebler; **Leibniz Institute for Prevention Research and Epidemiology (Germany)**: Claudia Boernhorst, Christoph Buck, Ronja Foraita, , Kathrin Günther, Antje Hebestreit, Hannah Jilani, Hermann Pohlabeln, Heike Schwarz, Ole Sprengeler, Barbara Thumann, Norman Wirsik, Maike Wolters, Achim Reineke, Claudia Brünings-Kuppe, Willempje Hummel-Bartenschlager, Ramona Siebels, Stefan Rach, Timm Intemann; **University of Milan (Italy)**: Claudio Galli (†), Patrizia Risé; **University of Pécs (Hungary)**: Dénes Molnar, Eva Kóvacs, Peter Nagy, Judit Répásy, Sarolta Stomfai, Eva Erhardt, Regina Heidinger-Felso;; **Universidad de Zaragoza (Spain)**: Luis A. Moreno, Silvia Bel-Serrat, Mª Pilar De Miguel-Etayo, Juan Fernández-Alvira, Esther Gonzalez-Gil, Luis Gracia-Marco, Iris Iglesia Altaba, Idoia Labayen, Maria Isabel Mesana-Graffe, Alba Mª Santaliestra-Pasías, Azahara Iris Rupérez Cano, Isabel Iguacel Azorín, Maria Luisa Miguel Berges, Natalia Giménez Legarre, Alelí Ayala Marín, Gloria Pérez Gimeno.
